# Cloning and expression profiling of the *VLDLR *gene associated with egg performance in duck (*Anas platyrhynchos*)

**DOI:** 10.1186/1297-9686-43-29

**Published:** 2011-08-05

**Authors:** Cui Wang, Shi-jun Li, Wen-hua Yu, Qing-wu Xin, Chuang Li, Yan-ping Feng, Xiu-li Peng, Yan-zhang Gong

**Affiliations:** 1 Key Laboratory of Agricultural Animal Genetics, Breeding, and Reproduction of Ministry of Education, Huazhong Agricultural University, Wuhan, Hubei 430070, PR China

## Abstract

**Background:**

The very low density lipoprotein receptor gene (*VLDLR*), a member of the low density lipoprotein receptor (*LDLR*) gene family, plays a crucial role in the synthesis of yolk protein precursors in oviparous species. Differential splicing of this gene has been reported in human, rabbit and rat. In chicken, studies showed that the VLDLR protein on the oocyte surface mediates the uptake of yolk protein precursors into growing oocytes. However, information on the *VLDLR *gene in duck is still scarce.

**Methods:**

Full-length duck *VLDLR *cDNA was obtained by comparative cloning and rapid amplification of cDNA ends (RACE). Tissue expression patterns were analysed by semi-quantitative reverse-transcription polymerase chain reaction (RT-PCR). Association between the different genotypes and egg performance traits was investigated with the general linear model (GLM) procedure of the SAS^® ^software package.

**Results:**

In duck, two *VLDLR *transcripts were identified, one transcript (variant-a) containing an O-linked sugar domain and the other (variant-b) not containing this sugar domain. These transcripts share ~70 to 90% identity with their counterparts in other species. A phylogenetic tree based on amino acid sequences showed that duck VLDLR proteins were closely related with those of chicken and zebra finch. The two duck *VLDLR *transcripts are differentially expressed i.e. *VLDLR-a *is mainly expressed in muscle tissue and *VLDLR-b *in reproductive organs. We have localized the duck *VLDLR *gene on chromosome Z. An association analysis using two completely linked SNP sites (T/C at position 2025 bp of the ORF and G/A in intron 13) and records from two generations demonstrated that the duck *VLDLR *gene was significantly associated with egg production (P < 0.01), age of first egg (P < 0.01) and body weight of first egg (P < 0.05).

**Conclusions:**

Duck and chicken *VLDLR *genes probably perform similar function in the development of growing oocytes and deposition of yolk lipoprotein. Therefore, *VLDLR *could be a candidate gene for duck egg performance and be used as a genetic marker to improve egg performance in ducks.

## Background

The very low density lipoprotein receptor (VLDLR), a member of the LDL receptor family [1], is an important multifunctional receptor. Apart from mediating the metabolism of triglycerides, it is well documented that VLDLR also takes part in a range of cellular processes including cell proliferation, migration and differentiation, etc [2].

The *VLDLR *gene was firstly isolated from a rabbit heart cDNA library and later cloned in chicken, human, mouse, cattle and monkey and its structure in these different species was elucidated in great detail [3-8]. Similar to the *LDLR *gene, the *VLDLR *gene contains five functional domains: (i) multiple cysteine-rich repeats constituting the amino-terminal ligand-binding domain; (ii) an epidermal growth factor (EGF) precursor homologous domain; (iii) an O-linked sugar domain; (iv) a transmembrane domain; and (v) a cytoplasmic domain with a FDNPVY sequence [9]. Although the structural features of each domain of the VLDLR and LDLR proteins share some striking homologies, they differ in the number of cysteine-rich repeat sequences present in the ligand-binding domain i.e. VLDLR has eight cysteine-rich repeats and LDLR, only has seven [10,11]. The O-linked sugar domain is a serine and threonine rich domain that corresponds to exon 16 in the *VLDLR *gene and its differential splicing has been described in human, rat, rabbit and cattle [7,12-15].

Chicken VLDLR, also named oocyte vitellogenesis receptor (OVR) or vitellogenin receptor (VTGR), mediates the absorption of yolk protein precursors from plasma very low density lipoprotein and vitellogenin. Bujo et al. (1994) detected a point mutation (G/C) at position 2177 bp of the chicken *VLDLR *cDNA (mutation named "restricted ovulation" or *RO*) and showed that the mutant had a reduced egg production [16-18]. Subsequently, it was shown that VLDLR has a key role on chicken reproduction, including the development of oocytes and yolk lipoprotein deposition [19,20]. Recently, a study in zebra finch suggested that *VLDLR *mRNA expression was pivotal for reproduction in oviparous species [21].

Duck is an important agricultural poultry species for the production of eggs and meat. However, egg performance of some local duck breeds remains low and could benefit from genetic improvement. Marker-assisted selection is based on the association between DNA variation and genes that control a trait of interest and has become an important approach towards improving production traits in animal breeding. In chicken and zebra finch, the *VLDLR *gene has been reported to play a key role in reproduction and could represent a functional candidate gene for egg performance. Since little was known on its structure and role in duck, we have cloned the full length duck *VLDLR *gene, analysed its expression profile in twelve different tissues and investigated its association with duck egg performance using SNP located within the gene.

## Materials and methods

### Ducks, tissue and data collection

Three healthy female ducks (aged 20 weeks) were selected from the second generation of the white *Lianchen*g × white *Kaiya *cross, and all the ducks were reared under normal management conditions. All animal procedures were performed according to protocols approved by the Biological Studies Animal Care and Use Committee of Hubei Province, PR China. Twelve different tissues were sampled from each duck, including heart, liver, spleen, lung, kidney, muscle, brain, adipose tissue, intestine, pituitary gland, ovary and oviduct, immediately frozen in liquid nitrogen and stored at -80°C until total RNA extraction.

Ducks of the second (n = 350) and third (n = 251) generations of white *Liancheng *× white *Kaiya *cross were provided by the Hankou Jingwu Industry Garden Ltd.. The ducks were reared in cages in a semi-open house and subjected to conventional management conditions. Recorded traits included age of the first egg, body weight at age of first egg and egg production (during 210 days, 300 days and 360 days) of each individual duck. Egg characteristics were measured at day 295 to 300, and included egg weight, Haugh unit, egg index, percentage of yolk, percentage of albumen and shell strength [22].

### DNA isolation, RNA isolation and cDNA synthesis

Genomic DNA was extracted by the phenol-chloroform method from blood samples [23]. DNA concentration and quality were measured with the spectrophotometer ND-1000 (Nano-Drop, USA), and the concentrations were adjusted between 50 and 300 ng/μL. DNA samples were stored at 4°C until use for PCR reactions.

Total RNA was isolated from different tissues with Trizol reagent (Invitrogen, Carlsbad, CA, USA) according to the manufacturer's protocol. The quality of total RNA sample was evaluated by electrophoresis on 1.2% agarose gels stained with ethidium bromide and their concentrations were measured with the spectrophotometer ND-1000 (Nano-Drop, USA). cDNA was obtained by reverse transcription polymerase chain reaction (RT-PCR) from 1 μg of DNase-treated (TOYOBO CO., DNaseI) total RNA according to the M-MLV reverse transcriptase kit (TOYOBO, Japan) at 42°C.

### Molecular cloning and sequence analysis of duck *VLDLR*

Based on the conserved region between *Gallus gallus *(GI: 45382562) and *Anser anser *(GI: 148733616) *VLDLR *genes, a pair of primers (*VLDLR-F/VLDLR-R*) was designed to obtain partial duck *VLDLR *gene sequence (primers shown in Table 1). The PCR program included denaturation during 5 min at 94°C, followed by 32 cycles of 30 s at 94°C, 30s at 60°C, 30s at 72°C, and an extension step of 5 min at 72°C. The PCR products were cloned into the PEASY-T1 vector (TransGen Biotech) and sequenced commercially.

**Table 1:** Primers used in this study

Primers purpose	Primer name	Primer sequence (5'-3')	Product size (bp)	Tm (°C)
RT-PCR	VLDLR-F	ATGGCCAGGATCGTAGACTT	292	58.0
	VLDLR-R	TCATTTATCTGAGGAGCAGG		
3'- RACE	GSP-3F	ATATTTGAGGACCGTGTGTACTGGA	1280	68.0
3'- Nested	NGSP-3F	ACTGGATCTGAATTGGTTACCCT		60.0
5'-RACE	GSP-5R	TGCATCATTGAGGTTGTTTACTAGG		68.0
5'-Nested	NGSP-5R1	ACTGCTTCATTCTCTCCATCAATCC	1006	60.0
	NGSP-5R2	GCATTCATTTATGTTGCATTCCT	742	62.0
	NGSP-5R3	TTTCACCATCACATTTCCAGGAC	590	65.0
Expression profile	VLDLR-A	AAAGTATACCTGTGCATGTCC	268/178	60.0
	VLDLR-S	CATGAAGTAGCCAGCCACC		
Internal control	β-actin-A	AACTGGGATGACATGGAGAAGA	104	60.0
	β-actin-S	GGGTTCAGGGGAGCCTCTGT		
	VLDLR-F1	TGTTCCTTCCTCATCCTCTT		
Polymorphism	VLDLR-R1	CAGAACAAACTCATAGCTACC	315	56.5
	VLDLR-F2	CAGGACATGCACAGGTATACTT	168	56.0
	VLDLR-R2	TACCTCTGGAGCATGAAGGCTCAC		

Based on the partial cDNA sequence obtained from the above RT-PCR reaction, duck gene specific primers and cDNA-end RACE primers were designed to amplify the full-length cDNA sequence of duck *VLDLR *(primers shown in Table 1). 3'-RACE and 5'-RACE PCR were conducted with 10 mg of RNA isolated from ovary and the SMART™ RACE cDNA Amplification kit (Clontech Laboratories, CA, USA) according to the manufacturer's instructions. The PCR program included a denaturation step of 4 min at 94°C, followed by 35 cycles of 35 s at 94°C, 35 s at annealing temperature (Table 1), 30 s to 2 min at 72°C, and a final extension of 5 min at 72°C.

The 3'-RACE and 5'-RACE PCR products were gel-purified and cloned into the PEASY-T1 vector (TransGen Biotech), then commercially sequenced. The open reading frame (ORF) and the amino acid sequences were deduced using SeqMan (DNAstar). The phylograms were created by MEGA 4.0 Neighbor-Joining (NJ) software [24]. The second structure prediction was performed using online tools on the ExPASy website (http://cn.expasy.org/tools/).

### Expression profiling

To determine the tissue expression of the two type splice variants, semi-quantitative RT-PCR were carried out using total RNA from various duck tissues and a pair of primers (*VLDLR-A/VLDLR-S*) encompassing the region corresponding to the O-linked sugar region (Table 1). The PCR program included a denaturation step of 5 min at 94°C, followed by 35 cycles of 30 s at 94°C, 30s at 60°C, 30s at 72°C, and a final step of 5 min at 72°C. As control, a pair of primers (*β-actin-A/β-actin-S*) (Table 1) was used under the same conditions. PCR products were visualized on 1.5% agarose gels stained with ethidium bromide and visualized with ultraviolet light.

### SNP screening and genotyping

Two pairs of specific primers (*VLDLR-F1/VLDLR-R1 *and *VLDLR-F2/VLDLR-R2*, Table 1) were designed to screen single nucleotide polymorphisms (SNP). Twelve DNA samples from the second generation ducks were amplified and sequenced. The obtained sequences were aligned by SeqMan (DNAStar software) to screen SNP based on the differences between sequences. The restriction endonuclease sites that harboured an SNP were detected with the primer premier 5.0 software to design the genotyping protocols. Genotyping of other individuals of the second and third generations were carried out by PCR-RFLP.

PCR for genotyping were performed in a volume of 15 μL consisting of 50-300 ng of genomic DNA, 1 × PCR buffer, 0.5 μM of each primer, 25 μM dNTP, 2.0 mM MgCl_2 _and 0.2 units Taq DNA polymerase (TransGen, Beijing, China), and ddH_2_O. PCR conditions were as follows: 4 min at 94°C, followed by 35 cycles of 30 s at 94°C, 30 s at 56°C, 35 s at 72°C, and a final step of 5 min at 72°C. Three μL of PCR product were digested overnight with 3 units of *Acc1/Rsa1 *(TaKaRa, Dalian, China) at 37°C, and then the digested products were visualized on 1.5% agarose gels stained with ethidium bromide and visualized with ultraviolet light, to record the genotype of each sample.

### Association analysis

The general linear model (GLM) procedures of SAS^® ^software package (SAS Inst. Inc., Cary NC, USA) was used to determine associations between the different genotypes with performance traits according to the following model, Y_ij_= μ+ S_i _+G_j_+ε_ij_, where Y_ij _is the observed value of different egg traits, μ is the population mean; S_i _and G_j _are the fixed effects of each generation and genotype, respectively, and ε_ij _is the random error. Values are considered significant at P < 0.05 and are presented as least square means ± standard error.

## Results and discussion

### Molecular cloning and sequence analysis of duck *VLDLR*

Using RACE and sequence matching techniques, a cDNA sequence covering the whole coding sequence was obtained from duck ovary. The cDNA consisted of 3450 nucleotides, containing an open reading frame (ORF) of 2553 bp, a 5'-terminal untranslated region (UTR) of 243 bp, and a 3'-terminal UTR of 654 bp including a TGA termination codon (nucleotides 2797~2799 bp), one putative polyadenylation consensus signal (AATAAA) and a poly (A) tail. The duck *VLDLR *nucleotide sequence shares ~70 to 90% similarity with its counterpart in other species including *Gallus gallus *(GI: 45382562), *Homo sapiens *(GI: 409425), *Macaca mulatta *(GI: 74136368), *Mus musculus *(GI: 609532), *Sus scrofa *(GI: 315506984), *Bos taurus *(GI: 31341853), *Danio rerio *(GI: 169646704), *Oryctolagus cuniculus *(GI: 126723672) and *Taeniopygia guttata *(GI: 224091307). However, unexpectedly we found that the duck *VLDLR *cDNA lacked approximately 90 nucleotides compared to the rabbit *VLDLR *cDNA. Differential splicing of *VLDLR *mRNA has been detected in rabbit, human, mouse and cattle and results from the deletion of the same region [7,12-15]. To confirm that a 90-bp deletion also occurred in duck, RT-PCR was carried out with total RNA from heart and a pair of primers flanking the deletion was designed. Two bands of 268 and 178 bp were amplified and cloned into T-vector and sequenced, which showed that the 268-bp fragment contained the additional 90-bp sequence. Thus, in duck, two *VLDLR *splice variants are present in heart, one (VLDLR-a) with an O-linked sugar domain and the other (VLDLR-b) without.

**Figure 1:**
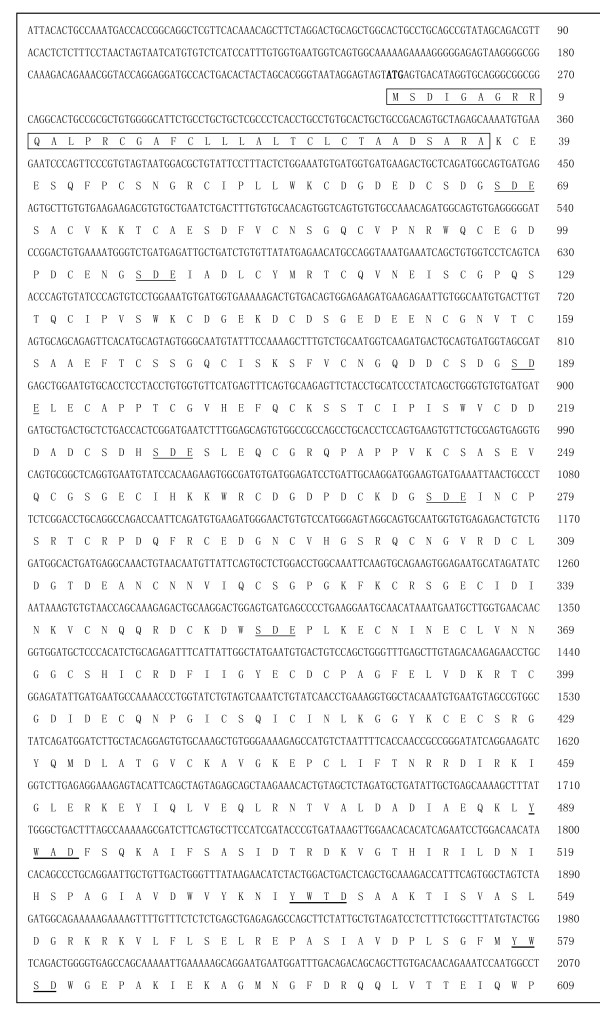
**Nucleotide and deduced amino acid sequences of the cDNA encoding the duck *VLDLR***. Letters in bold character indicate the start codon (ATG); the cleaved signal sequence is boxed at the N-terminus; different ligand binding motifs within the ligand domain are underlined; YWXD repeats are indicated by a thick underline.

**Figure 2:**
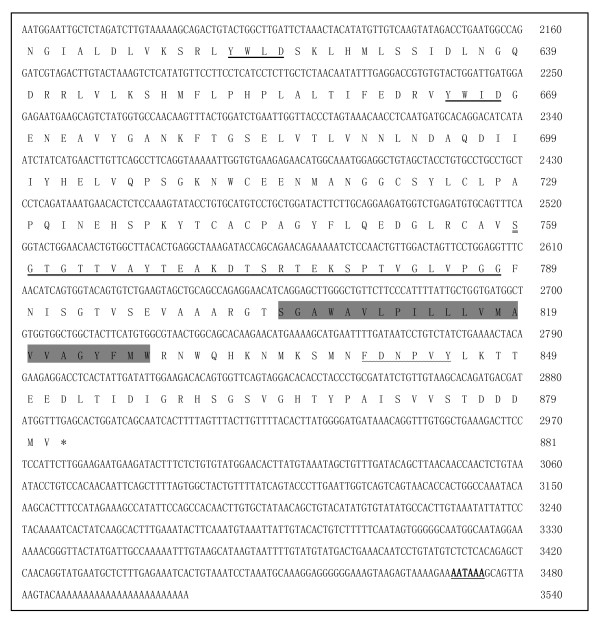
**Nucleotide and deduced amino acid sequences of the cDNA encoding the duck *VLDLR***. An asterisk indicates the stop codon (TGA) and the polyadenylation signal sequence (AATAAA) is underlined and in bold character; YWXD repeats are indicated by a thick underline; serine and threonine residues that correspond to the O-linked sugar domain are indicated by double underline; the 23 amino-acid putative transmembrane domain is shaded; the FDNPVY sequence targeting the receptor to coated pits is marked by a wavy underline.

The prediction results from the Swiss Institute of Bioinformatics software showed that the *VLDLR-a *(GenBank: JF950611) contained a 2643 bp ORF, and encoded a protein of 881 amino acids (aa) with an isoelectric point (pI) of 4.70 and calculated molecular mass (MW) of 96.73 kDa. The *VLDLR-b *(GenBank: JF950612) contained a 2553 bp ORF and encoded a protein of 851 aa with a pI of 4.69 and calculated MW of 93.74 kDa. Similar to the *LDLR *transcript, the *VLDLR-a *consists of five domains (Figure 1, 2): (i) six ligand binding motifs (S-D-E) and eight cysteine-rich repeats within the ligand binding domain; (ii) five YWXD motifs in the EGF precursor homology domain; (iii) an O-linked sugar domain with clustered serine and threonine residues; (iv) a 23-aa sequence in the putative transmembrane domain and (v) a FDNPVY sequence in the cytoplasmic domain at the C- terminus [3-7,9]. The *VLDLR-b *form lacks the O-linked sugar domain.

**Figure 3:**
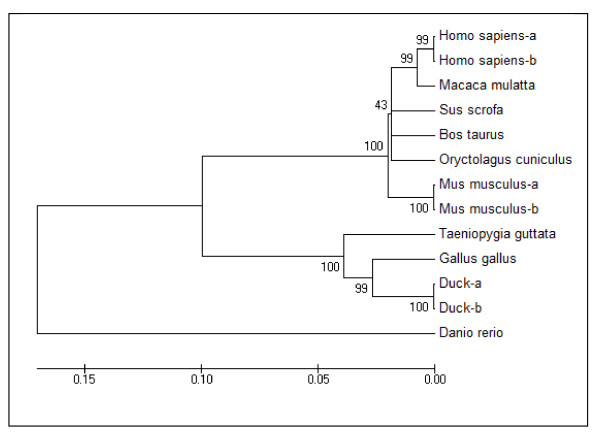
**The phylogenetic tree of duck VLDLR amino acid sequences with other eleven animal species**. The phylogenetic tree was constructed by the Neighbor-Joining (NJ) method of MEGA 4.0 using the deduced amino acid VLDLR sequence of duck and eleven other animal species; the number at the branches denotes the bootstrap majority consensus values on 1000 replicates; the branch lengths represent the relative genetic distance among these species.

To investigate the evolutionary origin of duck VLDLR, a phylogenetic tree showed in Figure 3 was constructed based on aa sequences of duck and eleven other animal species for which a complete aa sequence was available, including *Gallus gallus *(GI: 45382563), *Homo sapiens-a *(GI: 65301167), *Homo sapiens-b *(GI: 65301164), *Macaca mulatta *(GI: 74136369), *Sus scrofa *(GI: 315139195), *Mus musculus-a *(GI: 238637303), *Mus musculus-b *(GI: 238637305), *Oryctolagus cuniculus *(GI: 126723673) and *Bos taurus *(GI: 27806193), *Danio rerio *(GI: 169646705) and *Taeniopygia guttata *(GI: 224091308) [3,4,7,21,25-28]. Based on this analysis, three branches were obtained with duck, chicken and zebra finch belonging to one group indicating that duck VLDLR proteins are closer to chicken and zebra finch VLDLR than to those of the other species. This suggests that duck, chicken and zebra finch VLDLR probably perform similar functions.

### Expression profile

**Figure 4:**
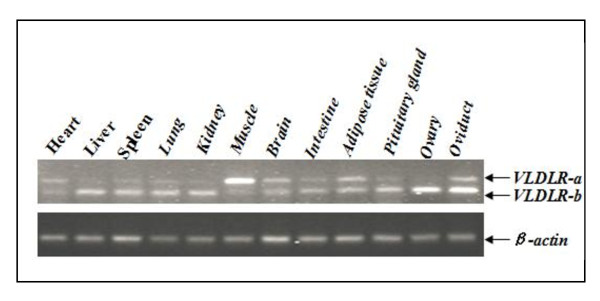
**Tissue expression profiles of the two splicing variants of duck *VLDLR *gene**. Tissue samples are heart, liver, spleen, lung, kidney, muscle, brain, intestine, adipose tissue, pituitary gland, ovary and oviduct from adult female ducks; *β-actin *is used as control.

To determine the tissue expression of the two duck splice variants, semi-quantitative RT-PCR was carried out with total RNA from twelve duck tissues and a pair of primers encompassing the region corresponding to the O-linked sugar region. As shown in Figure 4, both transcripts were expressed in nearly all the tissues tested from adult female ducks. The *VLDLR-a *was highly expressed in muscle tissue, while the *VLDLR-b *was predominantly expressed in ovary, oviduct, pituitary gland, liver, spleen, lung, kidney and intestine. Both transcripts are expressed at equivalent levels in heart, brain and adipose tissues.

In rabbit, the *VLDLR *transcript with the O-linked sugar region (type-1 *VLDLR*) is the major transcript in heart and muscle, whereas the transcript for *VLDLR *lacking the O-linked sugar region (type-2 *VLDLR*) is preferentially expressed in non-muscle tissues, including cerebrum, cerebellum, kidney, spleen, adrenal gland, testis, ovary and uterus [15]. In cattle, the variant with the O-linked sugar domain is mainly expressed in heart, brain and skeletal muscle, while the non-O-glycosylated splice variant is found in all detected tissues [7]. In our study, a differential representation of the two splice variants was also observed, *VLDLR-a *was predominantly expressed in muscle tissue, and there was no obvious differential expression in heart and brain tissues. Considering that the differential expression of both *VLDLR *variants varies slightly among species and tissues, the roles of each isoform may differ. In addition, the fact that the transcript lacking the O-linked sugar region (*VLDLR-b*) and expressed in ovary emphasizes its specific role in the development of growing oocytes.

### SNP screening and genotyping

**Figure 5:**
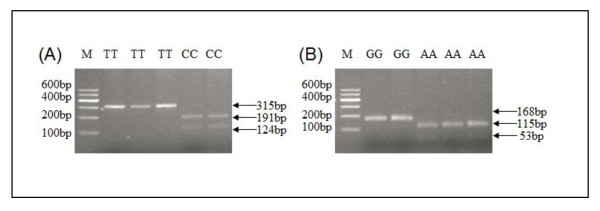
**(A) *AccI*-PCR-RFLP and (B) *RsaI*-PCR- RFLP genotyping of duck *VLDLR *gene**. TT (315 bp) and CC (191/124 bp) genotypes for the *Acc1 *site (A) and GG (168 bp) and AA (115/53 bp) genotypes for the *Rsa1 *site (B) were generated. The genotypes are shown at the top of each lane; M is marker1.

Alignment of the PCR sequences from different individuals detected two SNP in a fragment of 714 bp (compared to the reference chicken genome *VLDLR *sequence, the fragment covers intron 12 to intron 13 and their flanking region sequences, GenBank: HQ446851 and HQ446852). These two SNP were positioned at 231 bp for C/T and 633 bp for G/A. These two mutations at 231 bp (i.e. at position 2025 bp of the ORF) and 633 bp (i.e. in intron 13 (reference chicken genome DNA sequence)) were selected to carry out a PCR-RFLP polymorphism analysis with *AccI *and *Rsa1*, respectively. For the *Acc1 *site, the 315 bp (T allele) PCR product was digested into two 191 and 124 bp fragments (C allele) (Figure 5-A). For the *Rsa1 *site, the 168 bp (G allele) PCR product was digested into two 115 and 53 bp fragments (A allele) (Figure 5-B). Genotyping data showed that these two sites are in complete linkage, and only two homozygotes were detected in our testing population.

Since a resource population was used and under the hypothesis that the gene is located on an autosome, heterozygous individuals would be expected. Thus, the only explanation is that in duck, the *VLDLR *gene is located on the Z chromosome, which agrees with the location of the chicken and zebra finch *VLDLR *genes also on the Z chromosome. Human *VLDLR *gene is on chromosome 9, which has been shown to share extensive conserved synteny with chicken Z [5,29]. Recently, Ellegren (2010) reported that the chromosomal evolution of birds occurs at an unusually slow rate and many chromosomes have remained more or less intact during avian evolution [30]. Thus, based on the genotype disequilibrium and the fact that duck is closely related to chicken and zebra finch, we conclude that the duck *VLDLR *gene is most likely located on the Z chromosome.

### Association analysis of the duck *VLDLR *gene with egg performance

The association analysis demonstrated a significant association between the two haplotypes and egg production, age at the first egg and body weight at the first egg. Hens with haplotype AT had a higher egg production (210-day egg production (P < 0.0001), 300-day egg production (P = 0.0003), 360-day egg production (P = 0.0002)) and earlier age for starting laying (P = 0.0001). Hens with haplotype CG had a higher body weight at the first egg (P = 0.0277) (Table 2).

**Table 2:** Association of two haplotypes with duck egg performance

Haplotype	AT(219)	CG(382)	P value
210 day egg production (n)	79.18 ± 1.07^A^	73.18 ± 0.81^B^	< 0.0001**
300 day egg production (n)	158.46 ± 1.50^A^	151.58 ± 1.14^B^	0.0003**
360 day egg production (n)	211.48 ± 1.82^A^	202.77 ± 1.38^B^	0.0002**
Age at the first egg (d)	118.12 ± 1.04^A^	123.11 ± 0.78^B^	0.0001**
Body weight at the first egg (kg)	1.781 ± 0.015^a^	1.824 ± 0.012^b^	0.0277*
Egg weight (g)	65.41 ± 0.53	65.98 ± 0.42	0.4000
Haugh unit	80.99 ± 0.84	80.11 ± 0.65	0.4084
Egg index	1.345 ± 0.006	1.342 ± 0.005	0.6571
Percentage of yolk	0.310 ± 0.003	0.312 ± 0.003	0.6934
Percentage of albumen	0.544 ± 0.003	0.546 ± 0.002	0.5993
Shell strength (kgf·cm^2^)	4.471 ± 0.074	4.585 ± 0.057	0.2291

Note: within the same columns, values with different capital letters indicate extremely significant differences (P < 0.01) and values with different lower case letters indicate significant differences (P < 0.05); ** indicates an extremely significant association at P < 0.01 and * indicates a significant association at P < 0.05.

In chicken, a naturally occurring point mutation (G/C) at position 2177 bp in the *OVR *cDNA resulting in an unpaired cysteine residue, prevents the normal yolk protein precursors to be accumulated, and causes a reduction or cessation of egg laying [17,31,32]. In zebra finch, it has been reported that the expression of *VLDLR *mRNA plays a key role in determining inter-individual variation in reproductive phenotype (e.g. follicle or egg size) [21]. In duck, the detected polymorphism may affect *VLDLR *mRNA stability through unknown mechanisms, influencing its expression in ovary, and the development of the growing oocytes and yolk lipoprotein deposition. The association analysis also confirmed the crucial role of *VLDLR *on the development of yolk protein precursors in oviparous species.

## Conclusions

In conclusion, two variants of duck *VLDLR *transcripts were identified and characterized, and their tissue expression patterns were analysed. Two complete linked SNP were screened and an association with egg performance was detected using a two generations population. Our results suggest that duck *VLDLR *could be a candidate gene for duck egg performance and used as a genetic marker to improve this trait.

## Competing interests

The authors declare that they have no competing interests.

## Authors' contributions

CW carried out the study and drafted the manuscript. YZG contributed to the study design and helped in revising the manuscript. SJL and WHY participated in the collection of duck blood and the measurement of egg traits. QWX and CL collected data of recorded traits. YPF and XLP participated in the design of the study. All authors read and approved the final manuscript.
